# Supplementary material for: FastqPuri: high-performance preprocessing of RNA-seq data
Source: BMC Bioinformatics. 2019 May 3;20:226. doi: 10.1186/s12859-019-2799-0 (PMC6500068; doi:10.1186/s12859-019-2799-0)
Supplement: Supplementary file 2 — Archive of FastqPuri. Archive containing all files needed to install and run FastqPuri v1.0.6. Date stamp March 22, 2019. (GZ 47,819 kb) [file 12859_2019_2799_MOESM2_ESM.gz › FastqPuri-1.0.6/html/struct__node.html]

FastqPuri: \_node Struct Reference


FastqPuri

Public Attributes |
List of all members

\_node Struct Reference

Node structure: formed out of T\_ACGT pointers to Node structure.
More...

`#include <tree.h>`

Collaboration diagram for \_node:

[legend]

|  |  |
| --- | --- |
| Public Attributes | |
| struct \_node \* | children [T\_ACGT] |
|  | |

## Detailed Description

Node structure: formed out of T\_ACGT pointers to Node structure.

## Member Data Documentation

## ◆ children

struct \_node\* \_node::children[T\_ACGT]

T\_ACGT pointers to Node structure

---

The documentation for this struct was generated from the following file:

- include/tree.h


---

Generated on Mon Mar 19 2018 23:42:01 for FastqPuri by  

 1.8.14
